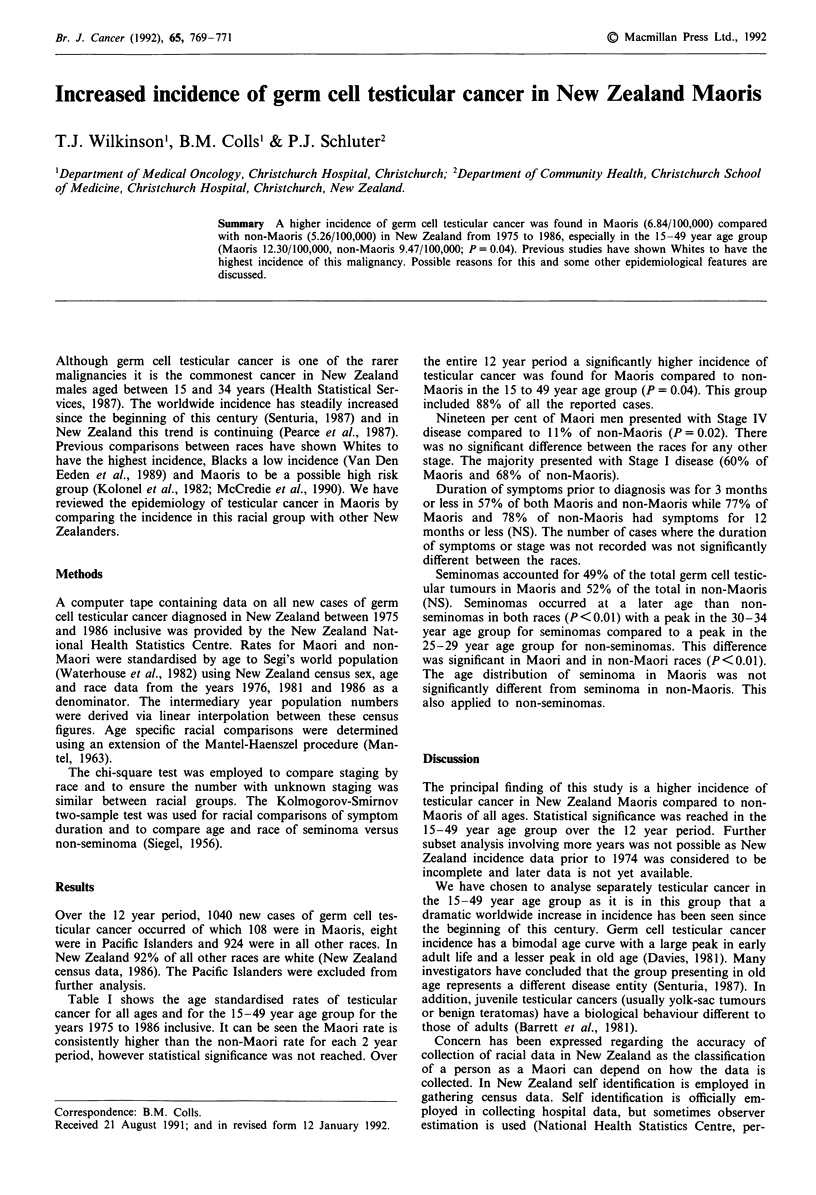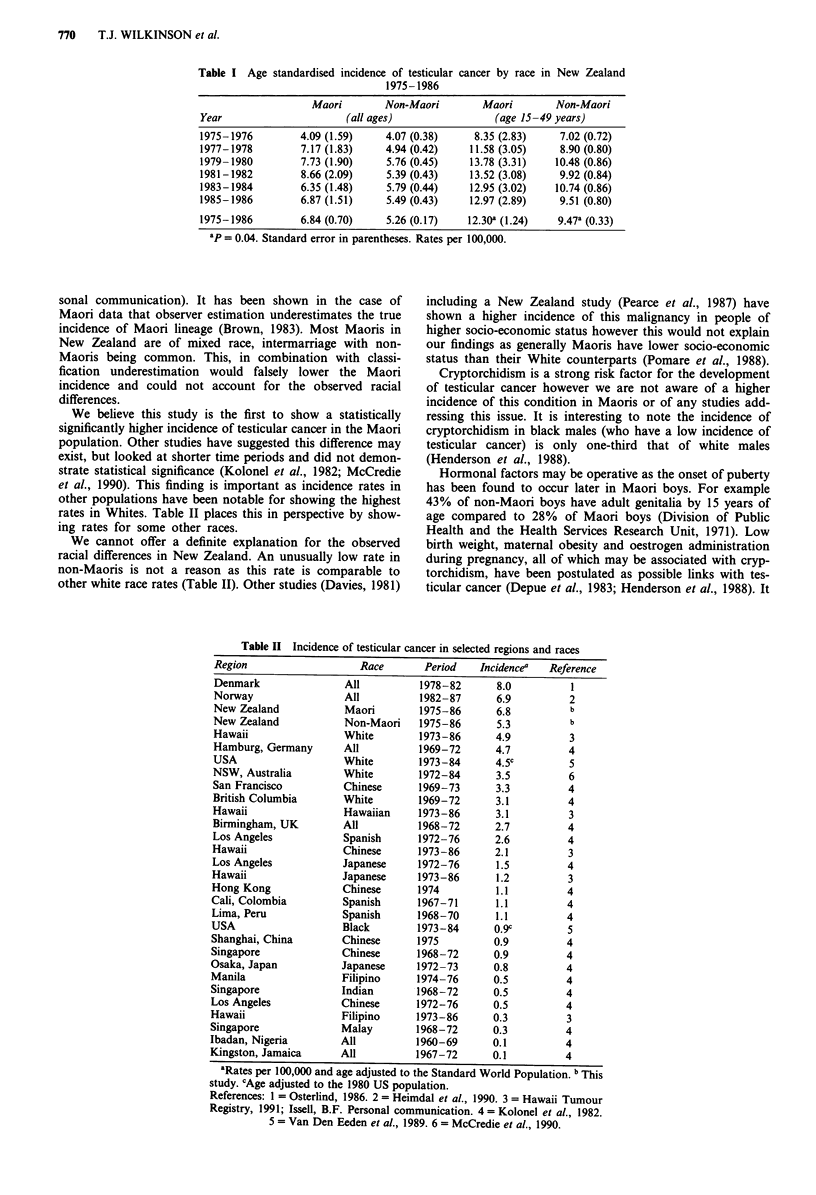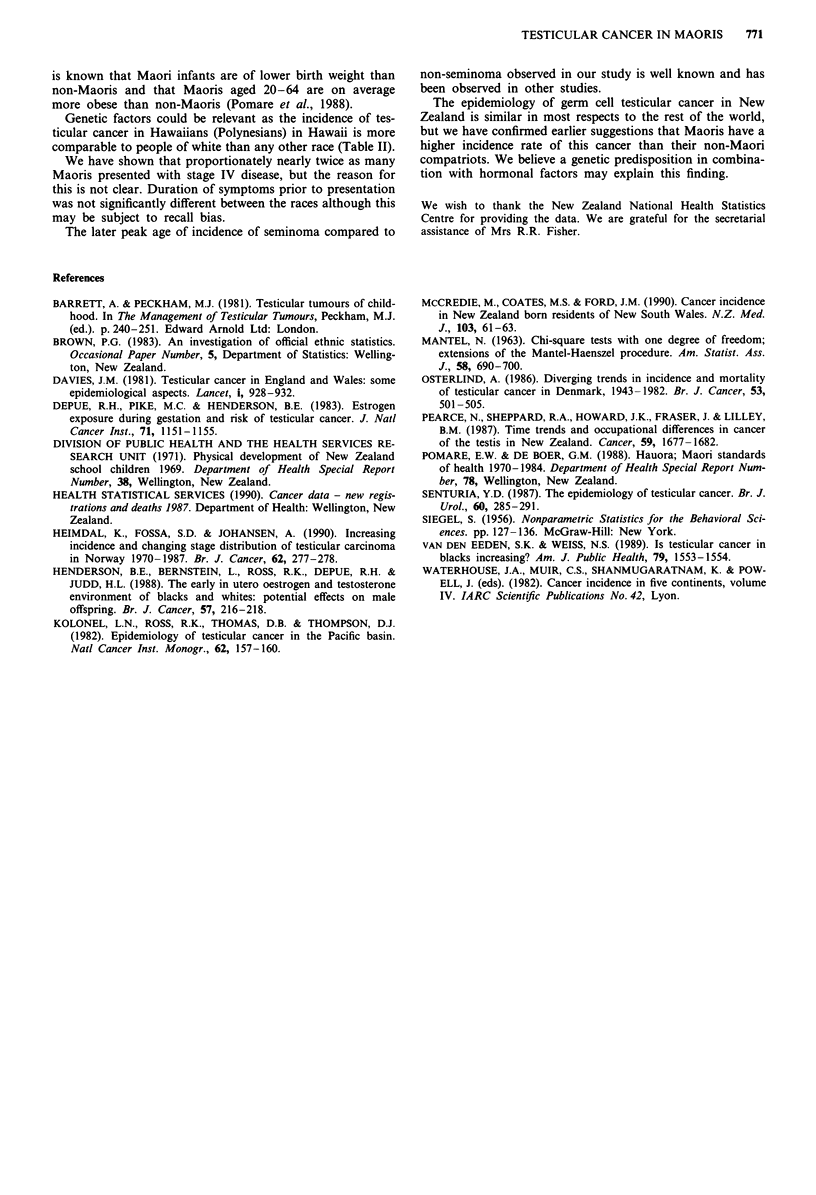# Increased incidence of germ cell testicular cancer in New Zealand Maoris.

**DOI:** 10.1038/bjc.1992.162

**Published:** 1992-05

**Authors:** T. J. Wilkinson, B. M. Colls, P. J. Schluter

**Affiliations:** Department of Medical Oncology, Christchurch Hospital, New Zealand.

## Abstract

A higher incidence of germ cell testicular cancer was found in Maoris (6.84/100,000) compared with non-Maoris (5.26/100,000) in New Zealand from 1975 to 1986, especially in the 15-49 year age group (Maoris 12.30/100,000, non-Maoris 9.47/100,000; P = 0.04). Previous studies have shown Whites to have the highest incidence of this malignancy. Possible reasons for this and some other epidemiological features are discussed.


Br. J. Cancer (1992), 65, 769–771 © Macmillan Press Ltd., 1992

### Increased incidence of germ cell testicular cancer in New Zealand Maoris

T.J. Wilkinson[1], B.M. Colls[1] & P.J. Schluter[2]

[1]*Department of Medical Oncology, Christchurch Hospital, Christchurch;* [2]*Department of Community Health, Christchurch School of Medicine, Christchurch Hospital, Christchurch, New Zealand.*

**Summary** A higher incidence of germ cell testicular cancer was found in Maoris (6.84/100,000) compared with non-Maoris (5.26/100,000) in New Zealand from 1975 to 1986, especially in the 15–49 year age group (Maoris 12.30/100,000, non-Maoris 9.47/100,000; $P = 0.04$). Previous studies have shown Whites to have the highest incidence of this malignancy. Possible reasons for this and some other epidemiological features are discussed.

Although germ cell testicular cancer is one of the rarer malignancies it is the commonest cancer in New Zealand males aged between 15 and 34 years (Health Statistical Services, 1987). The worldwide incidence has steadily increased since the beginning of this century (Senturia, 1987) and in New Zealand this trend is continuing (Pearce *et al.*, 1987). Previous comparisons between races have shown Whites to have the highest incidence, Blacks a low incidence (Van Den Eeden *et al.*, 1989) and Maoris to be a possible high risk group (Kolonel *et al.*, 1982; McCredie *et al.*, 1990). We have reviewed the epidemiology of testicular cancer in Maoris by comparing the incidence in this racial group with other New Zealanders.

### Methods

A computer tape containing data on all new cases of germ cell testicular cancer diagnosed in New Zealand between 1975 and 1986 inclusive was provided by the New Zealand National Health Statistics Centre. Rates for Maori and non-Maori were standardised by age to Segi's world population (Waterhouse *et al.*, 1982) using New Zealand census sex, age and race data from the years 1976, 1981 and 1986 as a denominator. The intermediary year population numbers were derived via linear interpolation between these census figures. Age specific racial comparisons were determined using an extension of the Mantel-Haenszel procedure (Mantel, 1963).

The chi-square test was employed to compare staging by race and to ensure the number with unknown staging was similar between racial groups. The Kolmogorov-Smirnov two-sample test was used for racial comparisons of symptom duration and to compare age and race of seminoma versus non-seminoma (Siegel, 1956).

### Results

Over the 12 year period, 1040 new cases of germ cell testicular cancer occurred of which 108 were in Maoris, eight were in Pacific Islanders and 924 were in all other races. In New Zealand 92% of all other races are white (New Zealand census data, 1986). The Pacific Islanders were excluded from further analysis.

Table I shows the age standardised rates of testicular cancer for all ages and for the 15–49 year age group for the years 1975 to 1986 inclusive. It can be seen the Maori rate is consistently higher than the non-Maori rate for each 2 year period, however statistical significance was not reached. Over the entire 12 year period a significantly higher incidence of testicular cancer was found for Maoris compared to non-Maoris in the 15 to 49 year age group ($P = 0.04$). This group included 88% of all the reported cases.

Nineteen per cent of Maori men presented with Stage IV disease compared to 11% of non-Maoris ($P = 0.02$). There was no significant difference between the races for any other stage. The majority presented with Stage I disease (60% of Maoris and 68% of non-Maoris).

Duration of symptoms prior to diagnosis was for 3 months or less in 57% of both Maoris and non-Maoris while 77% of Maoris and 78% of non-Maoris had symptoms for 12 months or less (NS). The number of cases where the duration of symptoms or stage was not recorded was not significantly different between the races.

Seminomas accounted for 49% of the total germ cell testicular tumours in Maoris and 52% of the total in non-Maoris (NS). Seminomas occurred at a later age than non-seminomas in both races ($P < 0.01$) with a peak in the 30–34 year age group for seminomas compared to a peak in the 25–29 year age group for non-seminomas. This difference was significant in Maori and in non-Maori races ($P < 0.01$). The age distribution of seminoma in Maoris was not significantly different from seminoma in non-Maoris. This also applied to non-seminomas.

### Discussion

The principal finding of this study is a higher incidence of testicular cancer in New Zealand Maoris compared to non-Maoris of all ages. Statistical significance was reached in the 15–49 year age group over the 12 year period. Further subset analysis involving more years was not possible as New Zealand incidence data prior to 1974 was considered to be incomplete and later data is not yet available.

We have chosen to analyse separately testicular cancer in the 15–49 year age group as it is in this group that a dramatic worldwide increase in incidence has been seen since the beginning of this century. Germ cell testicular cancer incidence has a bimodal age curve with a large peak in early adult life and a lesser peak in old age (Davies, 1981). Many investigators have concluded that the group presenting in old age represents a different disease entity (Senturia, 1987). In addition, juvenile testicular cancers (usually yolk-sac tumours or benign teratomas) have a biological behaviour different to those of adults (Barrett *et al.*, 1981).

Concern has been expressed regarding the accuracy of collection of racial data in New Zealand as the classification of a person as a Maori can depend on how the data is collected. In New Zealand self identification is employed in gathering census data. Self identification is officially employed in collecting hospital data, but sometimes observer estimation is used (National Health Statistics Centre, per-

Correspondence: B.M. Colls.
Received 21 August 1991; and in revised form 12 January 1992.

Table I Age standardised incidence of testicular cancer by race in New Zealand 1975-1986

| Year | Maori | Non-Maori | Maori | Non-Maori |
|---|---|---|---|---|
| | (all ages) | | (age 15-49 years) | |
| 1975-1976 | 4.09 (1.59) | 4.07 (0.38) | 8.35 (2.83) | 7.02 (0.72) |
| 1977-1978 | 7.17 (1.83) | 4.94 (0.42) | 11.58 (3.05) | 8.90 (0.80) |
| 1979-1980 | 7.73 (1.90) | 5.76 (0.45) | 13.78 (3.31) | 10.48 (0.86) |
| 1981-1982 | 8.66 (2.09) | 5.39 (0.43) | 13.52 (3.08) | 9.92 (0.84) |
| 1983-1984 | 6.35 (1.48) | 5.79 (0.44) | 12.95 (3.02) | 10.74 (0.86) |
| 1985-1986 | 6.87 (1.51) | 5.49 (0.43) | 12.97 (2.89) | 9.51 (0.80) |
| 1975-1986 | 6.84 (0.70) | 5.26 (0.17) | 12.30[a] (1.24) | 9.47[a] (0.33) |

[a]$P = 0.04$. Standard error in parentheses. Rates per 100,000.

sonal communication). It has been shown in the case of Maori data that observer estimation underestimates the true incidence of Maori lineage (Brown, 1983). Most Maoris in New Zealand are of mixed race, intermarriage with non-Maoris being common. This, in combination with classification underestimation would falsely lower the Maori incidence and could not account for the observed racial differences.

We believe this study is the first to show a statistically significantly higher incidence of testicular cancer in the Maori population. Other studies have suggested this difference may exist, but looked at shorter time periods and did not demonstrate statistical significance (Kolonel *et al.*, 1982; McCredie *et al.*, 1990). This finding is important as incidence rates in other populations have been notable for showing the highest rates in Whites. Table II places this in perspective by showing rates for some other races.

We cannot offer a definite explanation for the observed racial differences in New Zealand. An unusually low rate in non-Maoris is not a reason as this rate is comparable to other white race rates (Table II). Other studies (Davies, 1981) including a New Zealand study (Pearce *et al.*, 1987) have shown a higher incidence of this malignancy in people of higher socio-economic status however this would not explain our findings as generally Maoris have lower socio-economic status than their White counterparts (Pomare *et al.*, 1988).

Cryptorchidism is a strong risk factor for the development of testicular cancer however we are not aware of a higher incidence of this condition in Maoris or of any studies addressing this issue. It is interesting to note the incidence of cryptorchidism in black males (who have a low incidence of testicular cancer) is only one-third that of white males (Henderson *et al.*, 1988).

Hormonal factors may be operative as the onset of puberty has been found to occur later in Maori boys. For example 43% of non-Maori boys have adult genitalia by 15 years of age compared to 28% of Maori boys (Division of Public Health and the Health Services Research Unit, 1971). Low birth weight, maternal obesity and oestrogen administration during pregnancy, all of which may be associated with cryptorchidism, have been postulated as possible links with testicular cancer (Depue *et al.*, 1983; Henderson *et al.*, 1988). It

Table II Incidence of testicular cancer in selected regions and races

| Region | Race | Period | Incidence[a] | Reference |
|---|---|---|---|---|
| Denmark | All | 1978-82 | 8.0 | 1 |
| Norway | All | 1982-87 | 6.9 | 2 |
| New Zealand | Maori | 1975-86 | 6.8 | b |
| New Zealand | Non-Maori | 1975-86 | 5.3 | b |
| Hawaii | White | 1973-86 | 4.9 | 3 |
| Hamburg, Germany | All | 1969-72 | 4.7 | 4 |
| USA | White | 1973-84 | 4.5[c] | 5 |
| NSW, Australia | White | 1972-84 | 3.5 | 6 |
| San Francisco | Chinese | 1969-73 | 3.3 | 4 |
| British Columbia | White | 1969-72 | 3.1 | 4 |
| Hawaii | Hawaiian | 1973-86 | 3.1 | 3 |
| Birmingham, UK | All | 1968-72 | 2.7 | 4 |
| Los Angeles | Spanish | 1972-76 | 2.6 | 4 |
| Hawaii | Chinese | 1973-86 | 2.1 | 3 |
| Los Angeles | Japanese | 1972-76 | 1.5 | 4 |
| Hawaii | Japanese | 1973-86 | 1.2 | 3 |
| Hong Kong | Chinese | 1974 | 1.1 | 4 |
| Cali, Colombia | Spanish | 1967-71 | 1.1 | 4 |
| Lima, Peru | Spanish | 1968-70 | 1.1 | 4 |
| USA | Black | 1973-84 | 0.9[c] | 5 |
| Shanghai, China | Chinese | 1975 | 0.9 | 4 |
| Singapore | Chinese | 1968-72 | 0.9 | 4 |
| Osaka, Japan | Japanese | 1972-73 | 0.8 | 4 |
| Manila | Filipino | 1974-76 | 0.5 | 4 |
| Singapore | Indian | 1968-72 | 0.5 | 4 |
| Los Angeles | Chinese | 1972-76 | 0.5 | 4 |
| Hawaii | Filipino | 1973-86 | 0.3 | 3 |
| Singapore | Malay | 1968-72 | 0.3 | 4 |
| Ibadan, Nigeria | All | 1960-69 | 0.1 | 4 |
| Kingston, Jamaica | All | 1967-72 | 0.1 | 4 |

[a]Rates per 100,000 and age adjusted to the Standard World Population. [b] This study. [c]Age adjusted to the 1980 US population.
References: 1 = Osterlind, 1986. 2 = Heimdal *et al.*, 1990. 3 = Hawaii Tumour Registry, 1991; Issell, B.F. Personal communication. 4 = Kolonel *et al.*, 1982. 5 = Van Den Eeden *et al.*, 1989. 6 = McCredie *et al.*, 1990.

is known that Maori infants are of lower birth weight than non-Maoris and that Maoris aged 20–64 are on average more obese than non-Maoris (Pomare *et al.*, 1988).

Genetic factors could be relevant as the incidence of testicular cancer in Hawaiians (Polynesians) in Hawaii is more comparable to people of white than any other race (Table II).

We have shown that proportionately nearly twice as many Maoris presented with stage IV disease, but the reason for this is not clear. Duration of symptoms prior to presentation was not significantly different between the races although this may be subject to recall bias.

The later peak age of incidence of seminoma compared to non-seminoma observed in our study is well known and has been observed in other studies.

The epidemiology of germ cell testicular cancer in New Zealand is similar in most respects to the rest of the world, but we have confirmed earlier suggestions that Maoris have a higher incidence rate of this cancer than their non-Maori compatriots. We believe a genetic predisposition in combination with hormonal factors may explain this finding.

We wish to thank the New Zealand National Health Statistics Centre for providing the data. We are grateful for the secretarial assistance of Mrs R.R. Fisher.

### References

BARRETT, A. & PECKHAM, M.J. (1981). Testicular tumours of childhood. In *The Management of Testicular Tumours*, Peckham, M.J. (ed.). p. 240–251. Edward Arnold Ltd: London.

BROWN, P.G. (1983). An investigation of official ethnic statistics. *Occasional Paper Number*, **5**, Department of Statistics: Wellington, New Zealand.

DAVIES, J.M. (1981). Testicular cancer in England and Wales: some epidemiological aspects. *Lancet*, **i**, 928–932.

DEPUE, R.H., PIKE, M.C. & HENDERSON, B.E. (1983). Estrogen exposure during gestation and risk of testicular cancer. *J. Natl Cancer Inst.*, **71**, 1151–1155.

DIVISION OF PUBLIC HEALTH AND THE HEALTH SERVICES RESEARCH UNIT (1971). Physical development of New Zealand school children 1969. *Department of Health Special Report Number*, **38**, Wellington, New Zealand.

HEALTH STATISTICAL SERVICES (1990). *Cancer data – new registrations and deaths 1987*. Department of Health: Wellington, New Zealand.

HEIMDAL, K., FOSSA, S.D. & JOHANSEN, A. (1990). Increasing incidence and changing stage distribution of testicular carcinoma in Norway 1970–1987. *Br. J. Cancer*, **62**, 277–278.

HENDERSON, B.E., BERNSTEIN, L., ROSS, R.K., DEPUE, R.H. & JUDD, H.L. (1988). The early in utero oestrogen and testosterone environment of blacks and whites: potential effects on male offspring. *Br. J. Cancer*, **57**, 216–218.

KOLONEL, L.N., ROSS, R.K., THOMAS, D.B. & THOMPSON, D.J. (1982). Epidemiology of testicular cancer in the Pacific basin. *Natl Cancer Inst. Monogr.*, **62**, 157–160.

MCCREDIE, M., COATES, M.S. & FORD, J.M. (1990). Cancer incidence in New Zealand born residents of New South Wales. *N.Z. Med. J.*, **103**, 61–63.

MANTEL, N. (1963). Chi-square tests with one degree of freedom; extensions of the Mantel-Haenszel procedure. *Am. Statist. Ass. J.*, **58**, 690–700.

OSTERLIND, A. (1986). Diverging trends in incidence and mortality of testicular cancer in Denmark, 1943–1982. *Br. J. Cancer*, **53**, 501–505.

PEARCE, N., SHEPPARD, R.A., HOWARD, J.K., FRASER, J. & LILLEY, B.M. (1987). Time trends and occupational differences in cancer of the testis in New Zealand. *Cancer*, **59**, 1677–1682.

POMARE, E.W. & DE BOER, G.M. (1988). Hauora; Maori standards of health 1970–1984. *Department of Health Special Report Number*, **78**, Wellington, New Zealand.

SENTURIA, Y.D. (1987). The epidemiology of testicular cancer. *Br. J. Urol.*, **60**, 285–291.

SIEGEL, S. (1956). *Nonparametric Statistics for the Behavioral Sciences*. pp. 127–136. McGraw-Hill: New York.

VAN DEN EEDEN, S.K. & WEISS, N.S. (1989). Is testicular cancer in blacks increasing? *Am. J. Public Health*, **79**, 1553–1554.

WATERHOUSE, J.A., MUIR, C.S., SHANMUGARATNAM, K. & POWELL, J. (eds). (1982). Cancer incidence in five continents, volume IV. *IARC Scientific Publications No. 42*, Lyon.